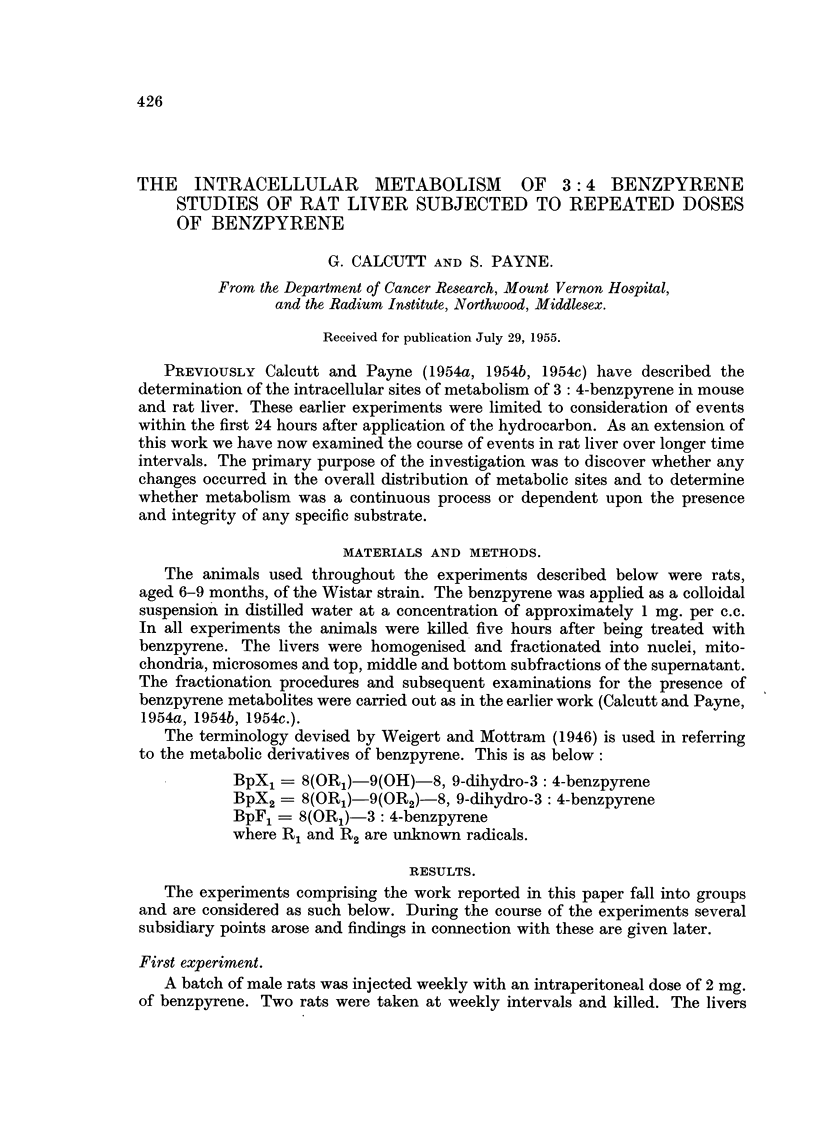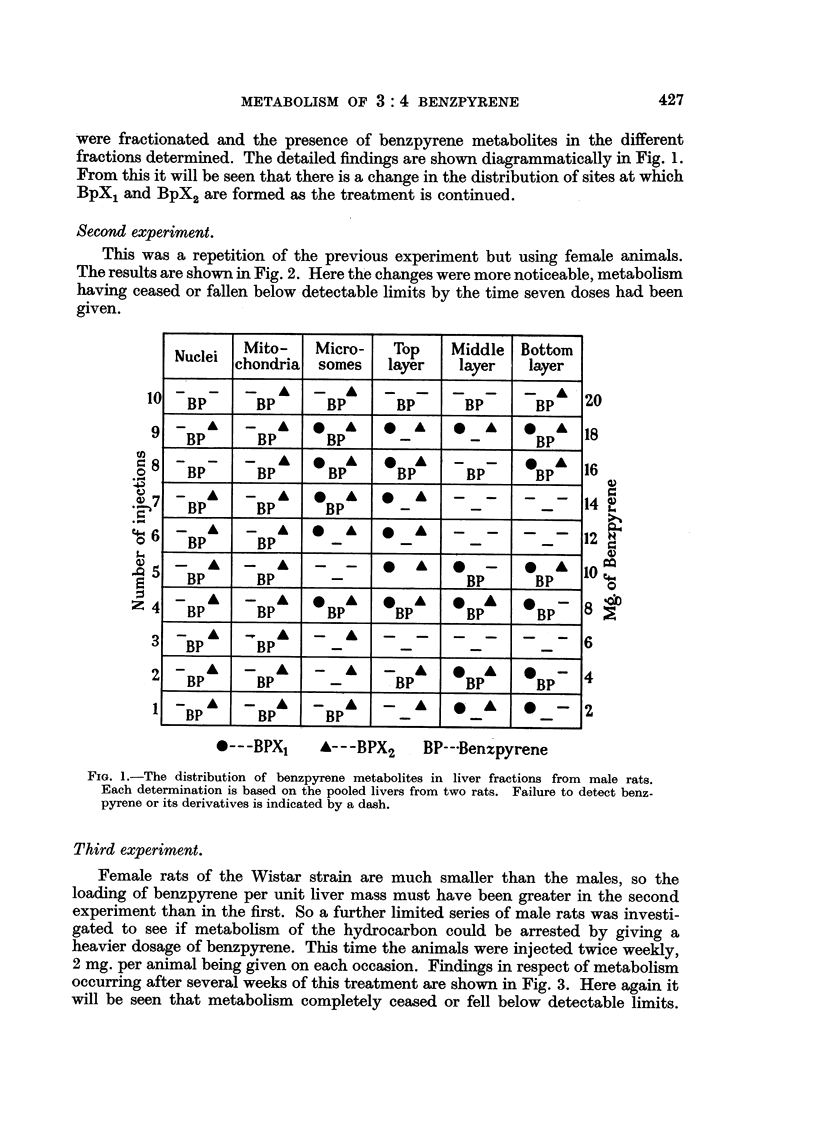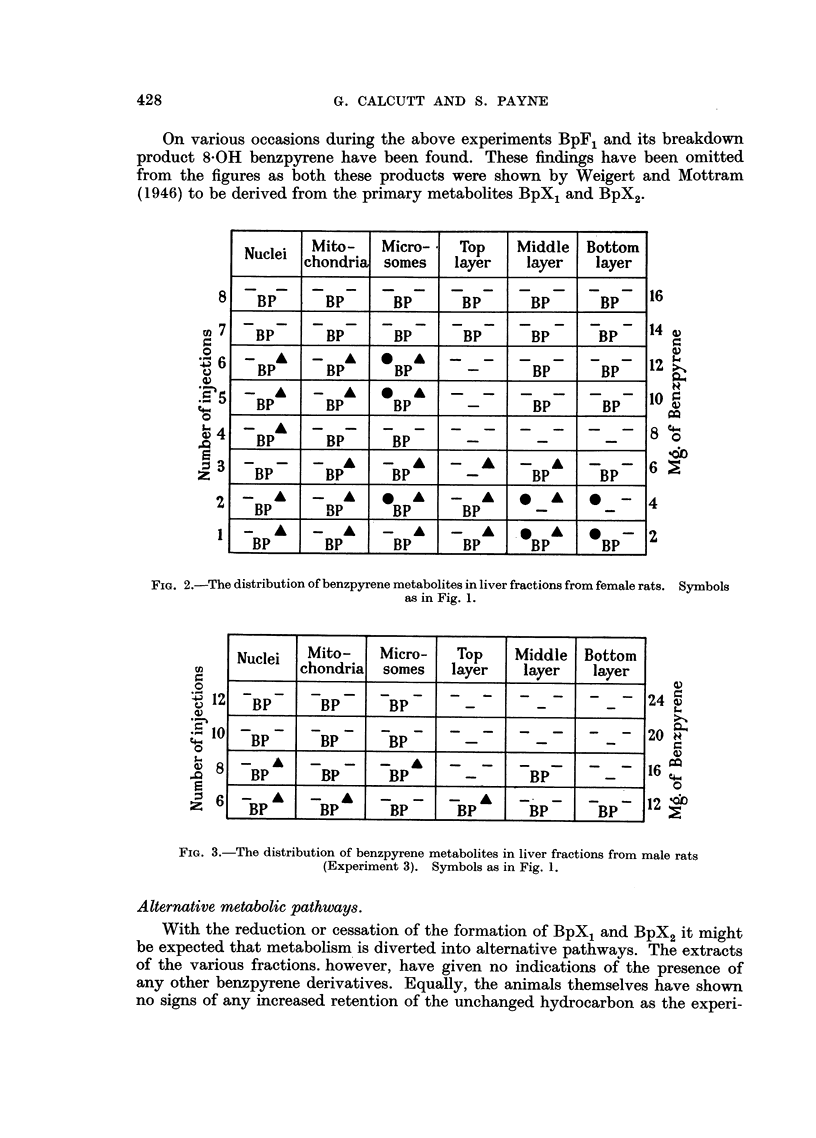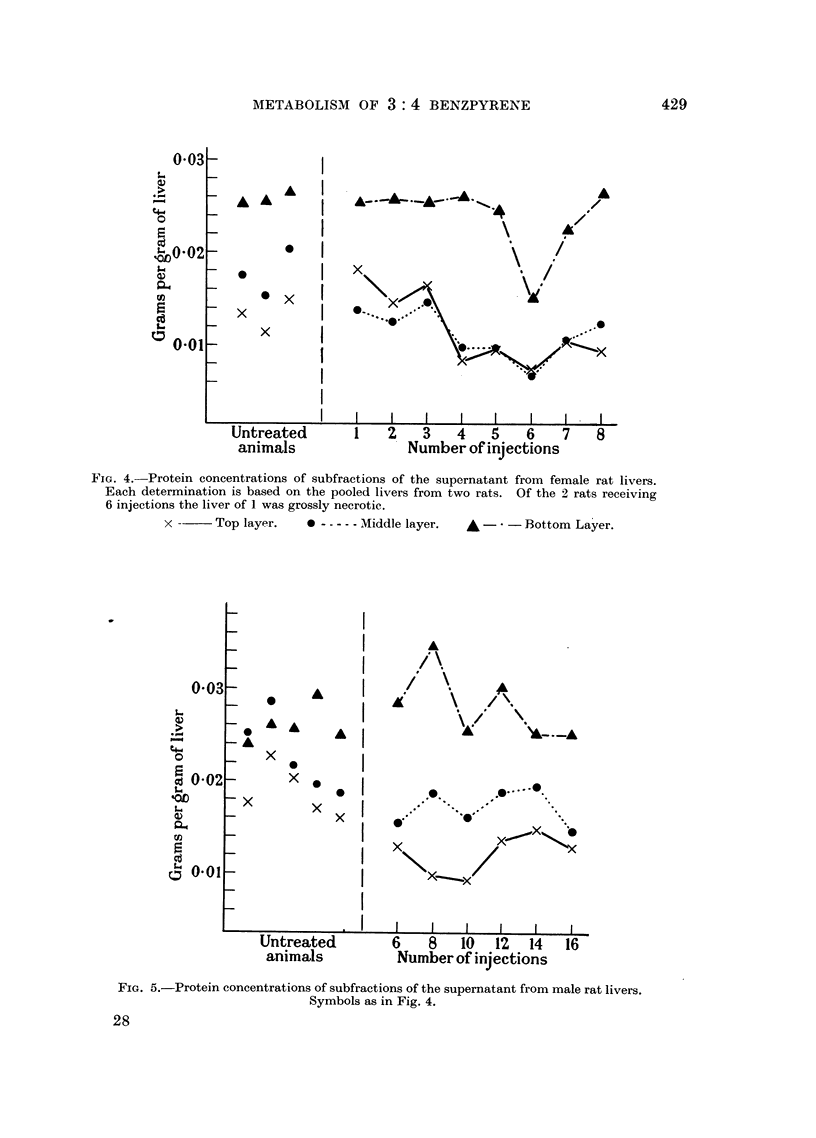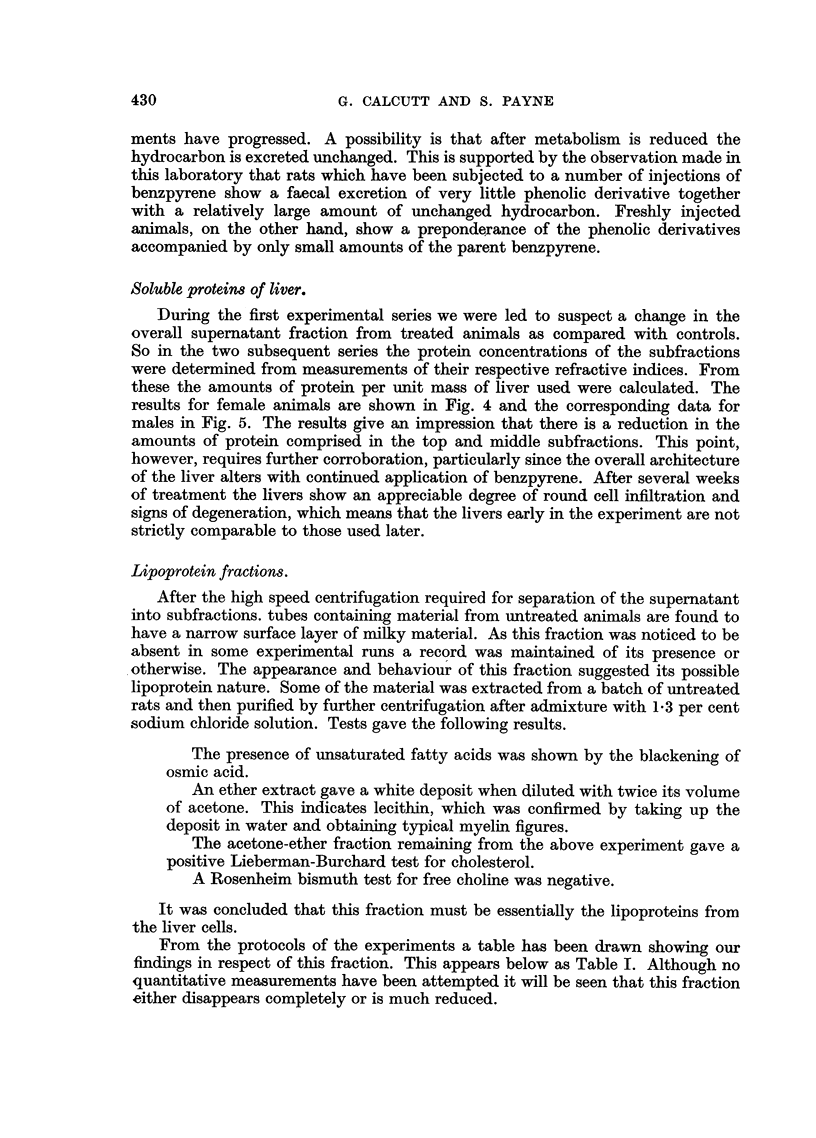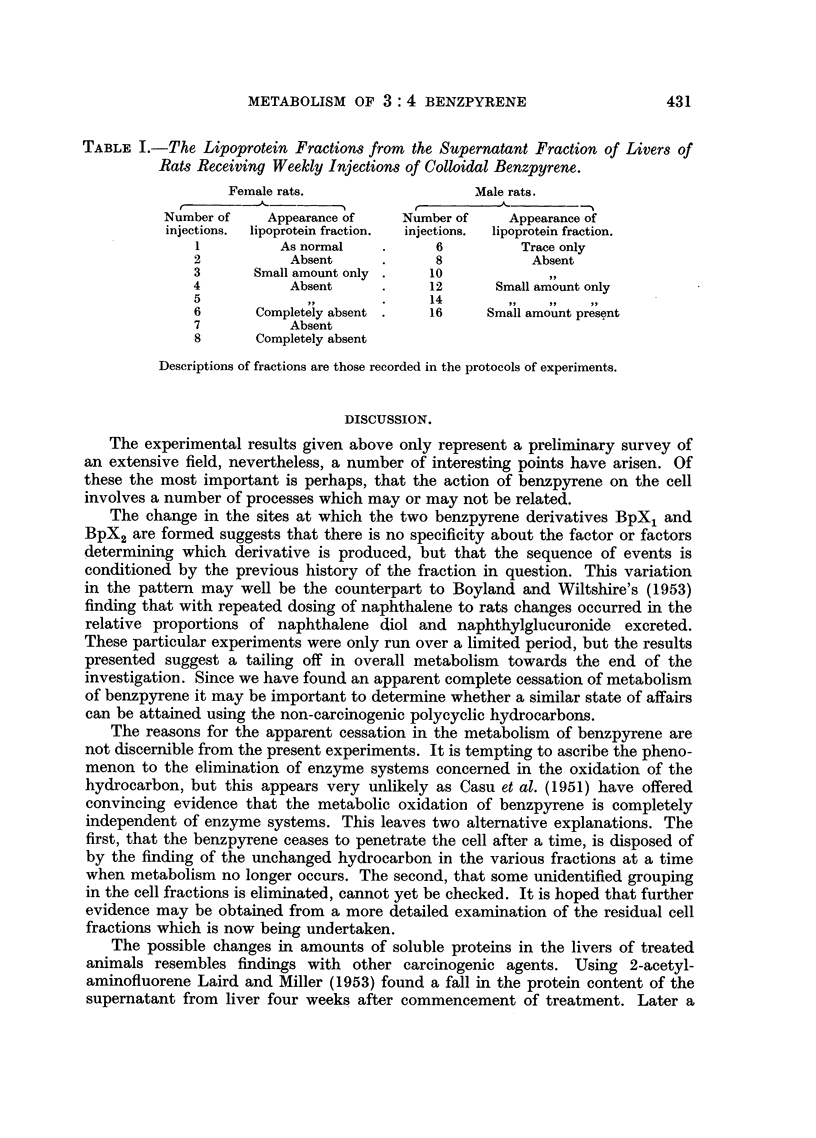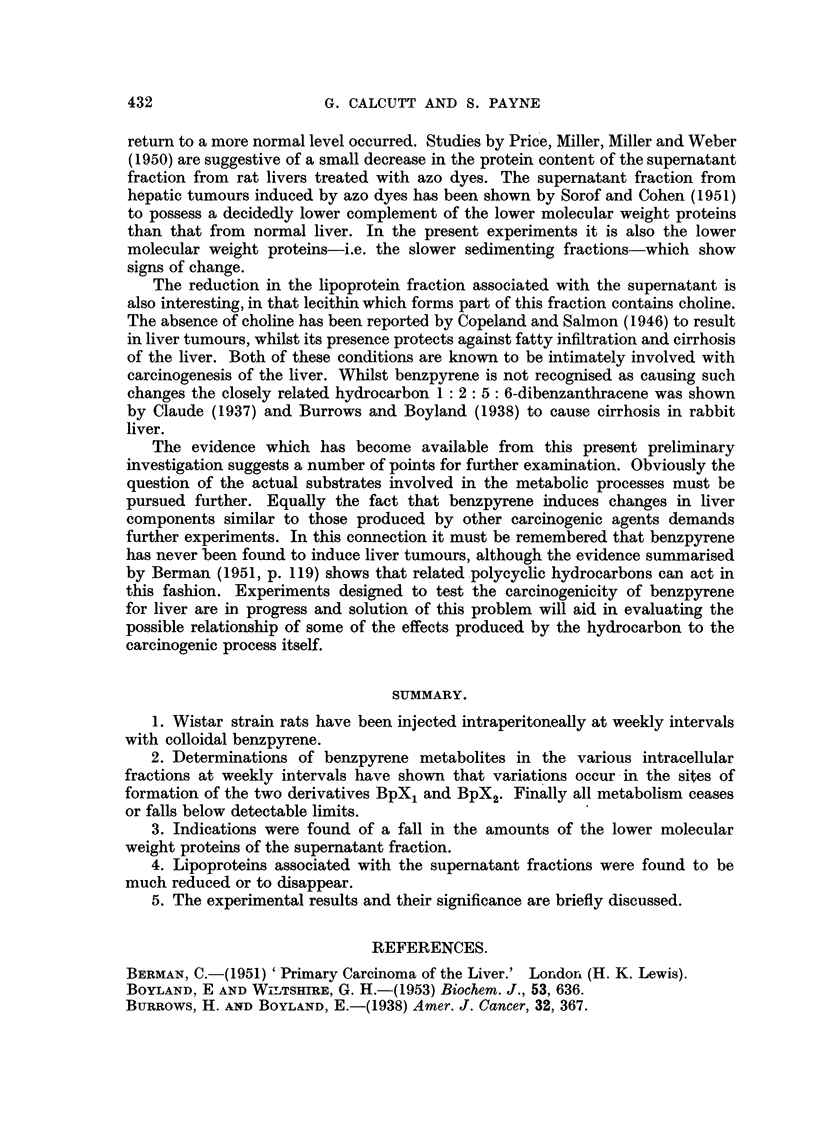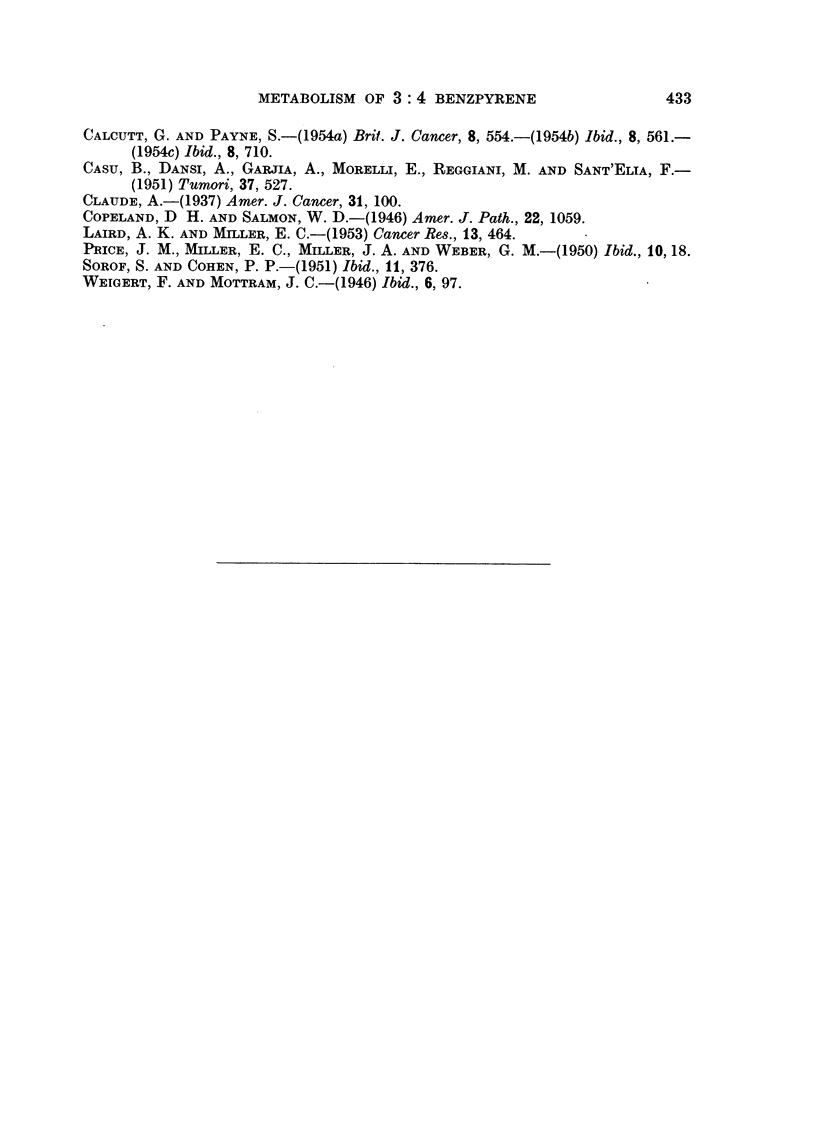# The Intracellular Metabolism of 3:4 Benzpyrene Studies of Rat Liver Subjected to Repeated Doses of Benzpyrene

**DOI:** 10.1038/bjc.1955.41

**Published:** 1955-09

**Authors:** G. Calcutt, S. Payne


### THE INTRACELLULAR METABOLISM OF 3:4 BENZPYRENE STUDIES OF RAT LIVER SUBJECTED TO REPEATED DOSES OF BENZPYRENE

G. CALCUTT AND S. PAYNE.

*From the Department of Cancer Research, Mount Vernon Hospital, and the Radium Institute, Northwood, Middlesex.*

Received for publication July 29, 1955.

PREVIOUSLY Calcutt and Payne (1954*a*, 1954*b*, 1954*c*) have described the determination of the intracellular sites of metabolism of 3 : 4-benzpyrene in mouse and rat liver. These earlier experiments were limited to consideration of events within the first 24 hours after application of the hydrocarbon. As an extension of this work we have now examined the course of events in rat liver over longer time intervals. The primary purpose of the investigation was to discover whether any changes occurred in the overall distribution of metabolic sites and to determine whether metabolism was a continuous process or dependent upon the presence and integrity of any specific substrate.

### MATERIALS AND METHODS.

The animals used throughout the experiments described below were rats, aged 6–9 months, of the Wistar strain. The benzpyrene was applied as a colloidal suspension in distilled water at a concentration of approximately 1 mg. per c.c. In all experiments the animals were killed five hours after being treated with benzpyrene. The livers were homogenised and fractionated into nuclei, mitochondria, microsomes and top, middle and bottom subfractions of the supernatant. The fractionation procedures and subsequent examinations for the presence of benzpyrene metabolites were carried out as in the earlier work (Calcutt and Payne, 1954*a*, 1954*b*, 1954*c*.).

The terminology devised by Weigert and Mottram (1946) is used in referring to the metabolic derivatives of benzpyrene. This is as below :

$BpX_1$ = 8($OR_1$)—9(OH)—8, 9-dihydro-3 : 4-benzpyrene

$BpX_2$ = 8($OR_1$)—9($OR_2$)—8, 9-dihydro-3 : 4-benzpyrene

$BpF_1$ = 8($OR_1$)—3 : 4-benzpyrene where $R_1$ and $R_2$ are unknown radicals.

### RESULTS.

The experiments comprising the work reported in this paper fall into groups and are considered as such below. During the course of the experiments several subsidiary points arose and findings in connection with these are given later.

### *First experiment.*

A batch of male rats was injected weekly with an intraperitoneal dose of 2 mg. of benzpyrene. Two rats were taken at weekly intervals and killed. The livers were fractionated and the presence of benzpyrene metabolites in the different fractions determined. The detailed findings are shown diagrammatically in Fig. 1. From this it will be seen that there is a change in the distribution of sites at which $BpX_1$ and $BpX_2$ are formed as the treatment is continued.

*Second experiment.*

This was a repetition of the previous experiment but using female animals. The results are shown in Fig. 2. Here the changes were more noticeable, metabolism having ceased or fallen below detectable limits by the time seven doses had been given.

FIG. 1.—The distribution of benzpyrene metabolites in liver fractions from male rats. Each determination is based on the pooled livers from two rats. Failure to detect benzpyrene or its derivatives is indicated by a dash.

*Third experiment.*

Female rats of the Wistar strain are much smaller than the males, so the loading of benzpyrene per unit liver mass must have been greater in the second experiment than in the first. So a further limited series of male rats was investigated to see if metabolism of the hydrocarbon could be arrested by giving a heavier dosage of benzpyrene. This time the animals were injected twice weekly, 2 mg. per animal being given on each occasion. Findings in respect of metabolism occurring after several weeks of this treatment are shown in Fig. 3. Here again it will be seen that metabolism completely ceased or fell below detectable limits.

On various occasions during the above experiments $BpF_1$ and its breakdown product 8·OH benzpyrene have been found. These findings have been omitted from the figures as both these products were shown by Weigert and Mottram (1946) to be derived from the primary metabolites $BpX_1$ and $BpX_2$.

FIG. 2.—The distribution of benzpyrene metabolites in liver fractions from female rats. Symbols as in Fig. 1.

FIG. 3.—The distribution of benzpyrene metabolites in liver fractions from male rats (Experiment 3). Symbols as in Fig. 1.

*Alternative metabolic pathways.*

With the reduction or cessation of the formation of $BpX_1$ and $BpX_2$ it might be expected that metabolism is diverted into alternative pathways. The extracts of the various fractions. however, have given no indications of the presence of any other benzpyrene derivatives. Equally, the animals themselves have shown no signs of any increased retention of the unchanged hydrocarbon as the experi-

FIG. 4.—Protein concentrations of subfractions of the supernatant from female rat livers. Each determination is based on the pooled livers from two rats. Of the 2 rats receiving 6 injections the liver of 1 was grossly necrotic.

× ——— Top layer. ● - - - - - Middle layer. ▲ — · — Bottom Layer.

FIG. 5.—Protein concentrations of subfractions of the supernatant from male rat livers. Symbols as in Fig. 4.

ments have progressed. A possibility is that after metabolism is reduced the hydrocarbon is excreted unchanged. This is supported by the observation made in this laboratory that rats which have been subjected to a number of injections of benzpyrene show a faecal excretion of very little phenolic derivative together with a relatively large amount of unchanged hydrocarbon. Freshly injected animals, on the other hand, show a preponderance of the phenolic derivatives accompanied by only small amounts of the parent benzpyrene.

*Soluble proteins of liver.*

During the first experimental series we were led to suspect a change in the overall supernatant fraction from treated animals as compared with controls. So in the two subsequent series the protein concentrations of the subfractions were determined from measurements of their respective refractive indices. From these the amounts of protein per unit mass of liver used were calculated. The results for female animals are shown in Fig. 4 and the corresponding data for males in Fig. 5. The results give an impression that there is a reduction in the amounts of protein comprised in the top and middle subfractions. This point, however, requires further corroboration, particularly since the overall architecture of the liver alters with continued application of benzpyrene. After several weeks of treatment the livers show an appreciable degree of round cell infiltration and signs of degeneration, which means that the livers early in the experiment are not strictly comparable to those used later.

*Lipoprotein fractions.*

After the high speed centrifugation required for separation of the supernatant into subfractions. tubes containing material from untreated animals are found to have a narrow surface layer of milky material. As this fraction was noticed to be absent in some experimental runs a record was maintained of its presence or otherwise. The appearance and behaviour of this fraction suggested its possible lipoprotein nature. Some of the material was extracted from a batch of untreated rats and then purified by further centrifugation after admixture with 1·3 per cent sodium chloride solution. Tests gave the following results.

The presence of unsaturated fatty acids was shown by the blackening of osmic acid.

An ether extract gave a white deposit when diluted with twice its volume of acetone. This indicates lecithin, which was confirmed by taking up the deposit in water and obtaining typical myelin figures.

The acetone-ether fraction remaining from the above experiment gave a positive Lieberman-Burchard test for cholesterol.

A Rosenheim bismuth test for free choline was negative.

It was concluded that this fraction must be essentially the lipoproteins from the liver cells.

From the protocols of the experiments a table has been drawn showing our findings in respect of this fraction. This appears below as Table I. Although no quantitative measurements have been attempted it will be seen that this fraction either disappears completely or is much reduced.

TABLE I.—*The Lipoprotein Fractions from the Supernatant Fraction of Livers of Rats Receiving Weekly Injections of Colloidal Benzpyrene.*

| Female rats. Number of injections. | Female rats. Appearance of lipoprotein fraction. | Male rats. Number of injections. | Male rats. Appearance of lipoprotein fraction. |
|---|---|---|---|
| 1 | As normal | 6 | Trace only |
| 2 | Absent | 8 | Absent |
| 3 | Small amount only | 10 | ,, |
| 4 | Absent | 12 | Small amount only |
| 5 | ,, | 14 | ,, ,, ,, |
| 6 | Completely absent | 16 | Small amount present |
| 7 | Absent | | |
| 8 | Completely absent | | |

Descriptions of fractions are those recorded in the protocols of experiments.

### DISCUSSION.

The experimental results given above only represent a preliminary survey of an extensive field, nevertheless, a number of interesting points have arisen. Of these the most important is perhaps, that the action of benzpyrene on the cell involves a number of processes which may or may not be related.

The change in the sites at which the two benzpyrene derivatives $BpX_1$ and $BpX_2$ are formed suggests that there is no specificity about the factor or factors determining which derivative is produced, but that the sequence of events is conditioned by the previous history of the fraction in question. This variation in the pattern may well be the counterpart to Boyland and Wiltshire's (1953) finding that with repeated dosing of naphthalene to rats changes occurred in the relative proportions of naphthalene diol and naphthylglucuronide excreted. These particular experiments were only run over a limited period, but the results presented suggest a tailing off in overall metabolism towards the end of the investigation. Since we have found an apparent complete cessation of metabolism of benzpyrene it may be important to determine whether a similar state of affairs can be attained using the non-carcinogenic polycyclic hydrocarbons.

The reasons for the apparent cessation in the metabolism of benzpyrene are not discernible from the present experiments. It is tempting to ascribe the phenomenon to the elimination of enzyme systems concerned in the oxidation of the hydrocarbon, but this appears very unlikely as Casu *et al.* (1951) have offered convincing evidence that the metabolic oxidation of benzpyrene is completely independent of enzyme systems. This leaves two alternative explanations. The first, that the benzpyrene ceases to penetrate the cell after a time, is disposed of by the finding of the unchanged hydrocarbon in the various fractions at a time when metabolism no longer occurs. The second, that some unidentified grouping in the cell fractions is eliminated, cannot yet be checked. It is hoped that further evidence may be obtained from a more detailed examination of the residual cell fractions which is now being undertaken.

The possible changes in amounts of soluble proteins in the livers of treated animals resembles findings with other carcinogenic agents. Using 2-acetylaminofluorene Laird and Miller (1953) found a fall in the protein content of the supernatant from liver four weeks after commencement of treatment. Later a return to a more normal level occurred. Studies by Price, Miller, Miller and Weber (1950) are suggestive of a small decrease in the protein content of the supernatant fraction from rat livers treated with azo dyes. The supernatant fraction from hepatic tumours induced by azo dyes has been shown by Sorof and Cohen (1951) to possess a decidedly lower complement of the lower molecular weight proteins than that from normal liver. In the present experiments it is also the lower molecular weight proteins—i.e. the slower sedimenting fractions—which show signs of change.

The reduction in the lipoprotein fraction associated with the supernatant is also interesting, in that lecithin which forms part of this fraction contains choline. The absence of choline has been reported by Copeland and Salmon (1946) to result in liver tumours, whilst its presence protects against fatty infiltration and cirrhosis of the liver. Both of these conditions are known to be intimately involved with carcinogenesis of the liver. Whilst benzpyrene is not recognised as causing such changes the closely related hydrocarbon 1 : 2 : 5 : 6-dibenzanthracene was shown by Claude (1937) and Burrows and Boyland (1938) to cause cirrhosis in rabbit liver.

The evidence which has become available from this present preliminary investigation suggests a number of points for further examination. Obviously the question of the actual substrates involved in the metabolic processes must be pursued further. Equally the fact that benzpyrene induces changes in liver components similar to those produced by other carcinogenic agents demands further experiments. In this connection it must be remembered that benzpyrene has never been found to induce liver tumours, although the evidence summarised by Berman (1951, p. 119) shows that related polycyclic hydrocarbons can act in this fashion. Experiments designed to test the carcinogenicity of benzpyrene for liver are in progress and solution of this problem will aid in evaluating the possible relationship of some of the effects produced by the hydrocarbon to the carcinogenic process itself.

### SUMMARY.

1. Wistar strain rats have been injected intraperitoneally at weekly intervals with colloidal benzpyrene.

2. Determinations of benzpyrene metabolites in the various intracellular fractions at weekly intervals have shown that variations occur in the sites of formation of the two derivatives $BpX_1$ and $BpX_2$. Finally all metabolism ceases or falls below detectable limits.

3. Indications were found of a fall in the amounts of the lower molecular weight proteins of the supernatant fraction.

4. Lipoproteins associated with the supernatant fractions were found to be much reduced or to disappear.

5. The experimental results and their significance are briefly discussed.

### REFERENCES.

Berman, C.—(1951) ' Primary Carcinoma of the Liver.' London (H. K. Lewis).
Boyland, E and Wiltshire, G. H.—(1953) *Biochem. J.*, **53**, 636.
Burrows, H. and Boyland, E.—(1938) *Amer. J. Cancer*, **32**, 367.

CALCUTT, G. AND PAYNE, S.—(1954*a*) *Brit. J. Cancer*, **8**, 554.—(1954*b*) *Ibid.*, **8**, 561.—(1954*c*) *Ibid.*, **8**, 710.
CASU, B., DANSI, A., GARJIA, A., MORELLI, E., REGGIANI, M. AND SANT'ELIA, F.—(1951) *Tumori*, **37**, 527.
CLAUDE, A.—(1937) *Amer. J. Cancer*, **31**, 100.
COPELAND, D H. AND SALMON, W. D.—(1946) *Amer. J. Path.*, **22**, 1059.
LAIRD, A. K. AND MILLER, E. C.—(1953) *Cancer Res.*, **13**, 464.
PRICE, J. M., MILLER, E. C., MILLER, J. A. AND WEBER, G. M.—(1950) *Ibid.*, **10**, 18.
SOROF, S. AND COHEN, P. P.—(1951) *Ibid.*, **11**, 376.
WEIGERT, F. AND MOTTRAM, J. C.—(1946) *Ibid.*, **6**, 97.